# Increased Plasma Levels of Triglyceride-Enriched Lipoproteins Associate with Systemic Inflammation, Lipopolysaccharides, and Gut Dysbiosis in Common Variable Immunodeficiency

**DOI:** 10.1007/s10875-023-01475-x

**Published:** 2023-03-30

**Authors:** Magnhild E. Macpherson, Tonje Skarpengland, Johannes R. Hov, Trine Ranheim, Beate Vestad, Tuva B. Dahl, Mai S. A. Fraz, Annika E. Michelsen, Kirsten B. Holven, Børre Fevang, Rolf K. Berge, Pål Aukrust, Bente Halvorsen, Silje F. Jørgensen

**Affiliations:** 1 Research Institute of Internal Medicine, Oslo University Hospital Rikshospitalet, Oslo, Norway; 2 Section of Clinical Immunology and Infectious Diseases, Oslo University Hospital Rikshospitalet, Oslo, Norway; 3 Institute of Clinical Medicine, University of Oslo, Oslo, Norway; 4 Norwegian PSC Research Center, Department of Transplantation Medicine, Division of Surgery, Inflammatory Diseases and Transplantation, Oslo University Hospital Rikshospitalet, Oslo, Norway; 5 Section of Gastroenterology, Department of Transplantation Medicine, Division of Surgery, Inflammatory Diseases and Transplantation, Oslo University Hospital, Oslo, Norway; 6 Department of Acute Medicine, Oslo University Hospital, Oslo, Norway; 7 Department of Nutrition, Institute for Basic Medical Sciences, University of Oslo, Oslo, Norway; 8 Norwegian National Advisory Unit On Familial Hypercholesterolemia, Oslo University Hospital Rikshospitalet, Oslo, Norway; 9 Centre for Rare Disorders, Oslo University Hospital, Oslo, Norway; 10 Department of Clinical Science, University of Bergen, N-5020 Bergen, Norway; 11 Department of Heart Disease, Haukeland University Hospital, N-5021 Bergen, Norway

**Keywords:** Triglycerides, VLDL, Lipids, LPS, CVID, Gut microbiota, Metabolism

## Abstract

**Purpose:**

Triglycerides (TG) and their major transport lipoprotein in the circulation (VLDL) appear to be related to inflammation. Patients with common variable immunodeficiency (CVID) have inflammatory complications associated with gut microbial dysbiosis. We hypothesized that CVID patients have disturbed TG/VLDL profiles associated with these clinical characteristics.

**Methods:**

We measured plasma concentrations of TGs, inflammatory markers, and lipopolysaccharide (LPS) in 95 CVID patients and 28 healthy controls. Additionally, in 40 CVID patients, we explored plasma lipoprotein profiling, fatty acid, gut microbial dysbiosis, and diet.

**Results:**

TG levels were increased in CVID patients as compared to healthy controls (1.36 ± 0.53 mmol/l versus 1.08 ± 0.56 [mean, SD], respectively, *P* = 0.008), particularly in the clinical subgroup “*Complications*,” characterized by autoimmunity and organ-specific inflammation, compared to “*Infection only*” (1.41 mmol/l, 0.71[median, IQR] versus [1.02 mmol/l, 0.50], *P* = 0.021). Lipoprotein profile analyses showed increased levels of all sizes of VLDL particles in CVID patients compared to controls. TG levels correlated positively with CRP (rho = 0.256, *P* = 0.015), IL-6 (rho = 0.237, *P* = 0.021), IL-12 (rho = 0.265, *P* = 0.009), LPS (*r* = 0.654, *P* = 6.59 × 10^−13^), CVID-specific gut dysbiosis index (*r* = 0.315, *P* = 0.048), and inversely with a favorable fatty acid profile (docosahexaenoic acid [rho =  − 0.369, *P* = 0.021] and linoleic acid [rho =  − 0.375, *P* = 0.019]). TGs and VLDL lipids did not appear to be associated with diet and there were no differences in body mass index (BMI) between CVID patients and controls.

**Conclusion:**

We found increased plasma levels of TGs and all sizes of VLDL particles, which were associated with systemic inflammation, LPS, and gut dysbiosis in CVID, but not diet or BMI.

**Supplementary Information:**

The online version contains supplementary material available at 10.1007/s10875-023-01475-x.

## Introduction

Common variable immunodeficiency (CVID) is the most prevalent symptomatic primary immunodeficiency in adults with a prevalence of 1:25,000 to 1:50,000 in Caucasians [1]. CVID patients are characterized by B cell defects with decreased serum levels of immunoglobulin (Ig) G as the most prominent feature, leading to recurrent airway infections with capsulated bacteria [2]. However, CVID constitutes a heterogeneous group of patients, in which 70–80% also have autoimmune and/or inflammatory complications, associated with increased morbidity and mortality [3–5]. These non-infectious complications are related to dysregulation of T cell and monocyte/macrophage function in addition to B cell pathology [6].

Several lines of evidence suggest an interaction between metabolic and inflammatory disturbances in various autoimmune and inflammatory disorders [7–9], representing a bidirectional pathogenic loop promoting disease progression. In line with this, we have recently showed similar features in subgroups of CVID patients with decreased levels and impaired anti-inflammatory function of high-density lipoprotein (HDL) cholesterol with normal levels of total cholesterol and low-density lipoprotein (LDL) cholesterol [10]. Additionally, we have identified an unfavorable fatty acid (FA) profile in CVID patients, which was associated with altered gut bacterial composition [10, 11]. Furthermore, we have shown that the gut bacteria–derived toxins and metabolites, such as lipopolysaccharides (LPS) and trimethylamine-N-oxide, are increased in CVID compared to healthy controls, and these molecules are also associated with systemic inflammation and gut microbial dysbiosis in CVID [3, 12]. However, gut microbial dysbiosis and related molecules have, so far, not been explored in relation to lipid metabolism in CVID.

Data on triglyceride (TG) levels in CVID are scarce [13]. Both TGs and their major transport lipoprotein in the circulation, very-low-density lipoprotein (VLDL), appear to be related to inflammation [14–17]. Through various mechanisms, systemic inflammation can enhance TG levels and some studies suggest that TG and VLDL may themselves promote inflammation [15, 18]. We hypothesized that CVID patients are characterized by disturbed TG and VLDL profiles and explored if TG and VLDL levels were related to clinical features, plasma markers of systemic inflammation and immune activation, gut dysbiosis, and systemic LPS levels, as well as diet, in the CVID group.

## Methods

### Participants and Procedures

CVID patients were recruited from the outpatient clinic at the Section of Clinical Immunology and Infectious Diseases, Oslo University Hospital Rikshospitalet. This study includes two different cohorts. The larger cohort consisted originally of 104 CVID patients recruited from November 2011 to December 2012 [12], but due to a technical issue (too low volume in nine samples), only 95 samples were analyzed for levels of TGs. This is referred to as the *Main cohort* in the study.

The smaller cohort of 40 CVID patients was recruited between October 2013 and October 2014 as previously described [19]. We have annotated this baseline cohort the *Subset cohort.* This cohort was a secondary data analysis of a randomized controlled trial where we utilized only the baseline samples (before randomization) [19]. In this cohort, we had, in addition to plasma samples, also detailed lipid profile, dietary registration data, and gut microbiota data. There was a considerable overlap between the *Main cohort* and *Subset cohort* in that 33 CVID patients were included in both cohorts. Of note, however, dietary registration data and gut microbiota data were not performed in the *Main cohort.*

For both cohorts, acute infection and acute exacerbation of inflammatory/autoimmune condition as well as treatment with immunomodulatory therapy, at the time of inclusion, were exclusion criteria. The *Subset cohort*, that included data on gut microbiota, had an additional exclusion criterion of no antibiotics in the last 12 weeks before inclusion. Details regarding exclusion criteria are given in Supplemental Methods.

For comparison, for both cohorts, we included 30 healthy controls on no regular medications. However, technical issues with the plasma samples from two healthy controls led to a reduced number of controls (*n* = 28) for the plasma analyses.

CVID was defined as decreased serum levels of IgG, IgA, and/or IgM by a minimum of two standard deviations below the mean for age, while excluding other causes of hypogammaglobulinemia [20]. Of note, both cohorts were included before the revised criteria from The European Society for Immunodeficiencies (ESID) were published in 2014 and 2019, but a retrospective analysis showed that 92 out 95 (97%) of the CVID patients, included in this study, would also have fulfilled the ESID 2019 criteria [21]. CVID subgroups were classified as “*Complications*” (i.e., presence of recurrent bacterial infections in the respiratory tract as well as one or more of the following non-infectious complications: splenomegaly, lymphoid hyperplasia, granulomas, enteropathy, organ-specific autoimmunity, autoimmune cytopenia, lymphoid interstitial pneumonitis/granulomatous lymphocytic interstitial lung disease, nodular regenerative hyperplasia, or lymphoma), or as “*Infection only*” (i.e., only recurrent bacterial infections in the respiratory tract and absence of the above-mentioned non-infectious complications), based on previously defined criteria. CVID enteropathy was defined as persistent diarrhea (> 3 months) after exclusion of gastrointestinal infection [12].

The Regional Committee for Medical and Research Ethics approved the study protocol (number 2013/1037 and 33,256). All study participants signed a written, informed consent. The work described in this article has been carried out in accordance with the Declaration of Helsinki.

### Blood Sampling Protocol

Non-fasting peripheral venous blood was drawn into sterile blood collection tubes with EDTA as anticoagulant. The tubes were immediately immersed in melting ice, centrifuged within 15 min at 2000* g* for 20 min to obtain platelet-poor plasma. Plasma was stored at − 80 °C in aliquots.

### Analyses of Inflammatory Parameters and Lipopolysaccharide

Plasma levels of soluble (s) CD14 and sCD25 were quantified in duplicates by enzyme immunoassays obtained from R&D Systems (Minneapolis, MN). Tumor necrosis factor (TNF) and interleukin (IL)-6, IL-8, and IL-12 were analyzed using a multiplex cytokine assay (Bio-Plex Human Cytokine Plex Panel; Bio-Rad Laboratories Inc., Hercules, CA), analyzed on a Multiplex Analyzer (Bio-Rad Laboratories) according to instructions from the manufacturer. High sensitivity C reactive protein (CRP) was analyzed on a MODULAR platform (Roche Diagnostics, Basel, Switzerland). LPS was analyzed by Limulus Amebocyte Lysate chromogenic assay (Lonza, Walkersville, MD) according to the manufacturer’s instructions, with the following modifications: samples were diluted tenfold to avoid interference with background colour and preheated to 68 °C for 10 min prior to analysis to dissolve immune complexes.

### Lipid Measurements

Plasma TG levels were measured enzymatically on a Hitachi 917 system (Roche Diagnosis GmbH, Mannheim, Germany) using the TG kits from Roche Diagnostics. Lipoprotein sub-classes were analyzed by using a metabolomics platform (Nightingale’s Biomarker Analysis Platform, Helsinki, Finland). The concentration and composition of different lipoprotein sub-classes were determined and classified into six VLDLs, one intermediate-density lipoprotein (IDL), and three LDLs (Supplemental Methods).

### Plasma FA Composition

The total FA composition was analyzed in EDTA-plasma, as previously described [22]. FA concentrations are expressed as percentages of total FAs by weight (wt %).

### Stool Collection and Analysis

Participants collected stool samples at home within 24 h prior to their hospital visit, or alternatively at the hospital, with a standardized collection device [23]. The stool samples were then transferred by the participants to stool collection tubes with Stool DNA Stabilizer (Stratec Biomedical, Birkenfeld, Germany) [24]. Samples were stored at minimum − 20 °C according to the manufacturer’s recommendations until DNA extraction. Bacterial DNA was extracted using the PSP Spin Stool DNA Plus Kit (Stratec) before being subjected to amplification of the 16S ribosomal RNA gene with dual-indexed barcodes according to an established protocol [25], followed by sequencing on an Illumina MiSeq (San Diego, CA; Supplemental Methods). In addition to alpha diversity measurements (Supplementary methods), we used the CVID-specific microbial dysbiosis index that consist of ten taxa that capture the dysbiosis in CVID [12, 19]. In detail, the CVID-specific microbial dysbiosis index was calculated for all samples as log (sum of the relative abundances of taxa upregulated in CVID) / (sum of the relative abundances of taxa reduced in CVID). For CVID this include (increased in CVID) *Bacilli*, *Dorea*, *Roseburia*, and *Gammaproteobacteria*, and (reduced in CVID) *Bifidobacterium*, *Odoribacteracea*, *Christensenellaceae*, *Blautia*, *Sutterella*, and *Desulfovibrionacea*.

### Food Frequency Questionnaire

CVID patients were asked to complete a self-administrated, validated Norwegian food frequency questionnaire (FFQ) designed to reflect dietary habits over the past year [26, 27]. The questionnaire offered multiple-choice alternatives and the opportunity to provide supplemental information regarding specific dietary restrictions or habits. It covered 180 food items and had serving size alternatives specified in household units, which were then converted to grams per day using software developed at the Institute for Nutrition Research, University of Oslo, Oslo, Norway.

### Statistical Analysis

Univariate analyses were performed using parametric (*t*-test) or non-parametric methods (Mann–Whitney *U*) for continuous variables, and Fisher’s exact test for categorical variables, as appropriate. Correlation analyses were performed using parametric (Pearson) or non-parametric (Spearman) tests as appropriate. Additionally, stepwise linear regression analysis was performed to adjust for age and sex. For the longitudinal data, we compared datasets from three different time points using the ANOVA test (parametric) or Friedman’s test (non-parametric). Calculations were performed in SPSS (version 24, IBM, NY). *P*-values are 2 sided and are considered significant at < 0.05.

## Results

### Patient Characteristics

The *Main cohort* included analyses of plasma samples from 95 CVID patients and 28 healthy controls. The *Subset cohort* consisted of plasma samples and stool samples from 40 CVID patients. Of these, 38 filled out the FFQ. Table 1 shows patients’ characteristics for both cohorts. There were no significant differences in age, body mass index (BMI), sex, or smoking habits between CVID patients and healthy controls in the *Main cohort*. All CVID patients, except two, were on Ig substitution (17 on Intravenous Ig [IVIG], 70 on subcutaneous Ig [SCIG], and six on both IVIG and SCIG). The mean serum IgG level in the CVID population was 7.7 g/l (SD 2.8 g/l, range 2.7–15.3 g/l).

**Table 1:** Background characteristics for CVID cohorts and healthy controls

	Main cohort			Subset cohort
	*CVID* *n* = *95*	*Controls* *n* = *28*	*P-value*^*a*^	*CVID* *n* = *40*
Age in years, mean ± SD	47 ± 15 [18–83]	42 ± 10 [28–65]	0.086^b^	50 ± 12 [21–69]
Female, *n* (%)	50 (53)	18 (64)	0.291^c^	25 (63)
BMI, mean ± SD	24 ± 4	24 ± 3	0.56^b^	26 ± 5
Smoking, *n* (%)	11 (11)	0(0)	0.068^c^	4 (10)
Infection only, *n* (%)	24 (25)	-	-	8 (20)
Complications	71 (75)			32 (80)
*Enteropathy*, *n (%)*	26 (27)			13 (33)
*Lymphoid hyperplasia*, *n (%)*	46 (48)			23 (58)
*Granulomas*, *n (%)*	17 (18)			6 (15)
*Organ-specific autoimmunity, n (%)*	20 (21)			8 (20)
*Autoimmune cytopenia*, *n (%)*	21 (22)			8 (20)

*BMI*, body mass index; *CVID*, common variable immunodeficiency

^a^*P*-value is given for CVID (*n* = 95) versus controls (*n* = 28)

^b^Mann–Whitney

^c^Fisher’s exact test

Infections only; no other complications to their immunodeficiency

### Increased Triglyceride Levels in CVID Patients

**Fig. 1:**
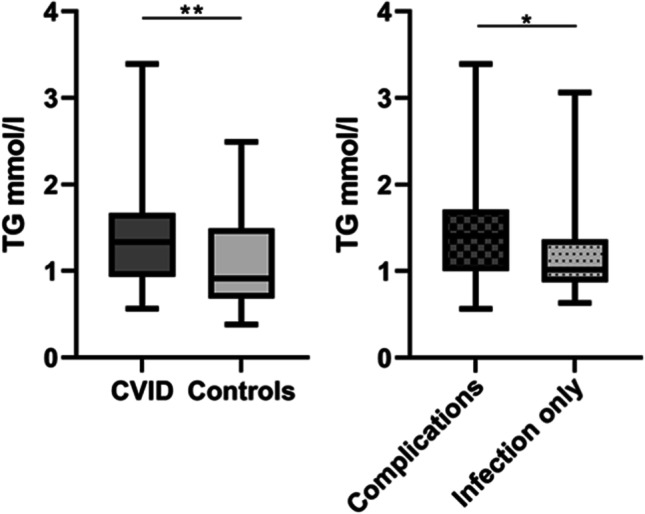
TG levels in CVID patients and controls including CVID subgroups. Plasma levels of TG in common variable immunodeficiency (CVID) patients and controls. The CVID cohort was further divided into two subgroups: Infection only and Complications (non-infectious). Results are given as boxes representing the 25th to 75th percentile with lines indicating median and whiskers min–max values;*P > 0.05, **P < 0.01, using *t*-test or Mann–Whitney test between groups. Data is from the Main cohort (95 CVID and 28 healthy controls)

As shown in Fig. 1A, TG levels were significantly increased in CVID patients (*n* = 95) as compared to healthy controls (*n* = 28) (1.36 ± 0.53 mmol/l vs. 1.08 ± 0.56 [mean, SD], respectively, *P* = 0.008). The *P*-value was attenuated but remained significant after adjusting for potential confounders such as age, BMI, and sex, *P* = 0.021. When applying the clinical CVID sub-classification of “*Infection only*” (*n* = 24) and “*Complications*” (*n* = 71), we found that TG levels were significantly increased in the clinical subgroup *Complications*, characterized by organ inflammation and autoimmunity (median 1.41 mmol/l, IQR 0.71), compared to “*Infection only*” (median 1.02 mmol/l, IQR 0.50), *P* = 0.021 (Fig. 1B). Of note, within the CVID group, TG levels did not correlate with BMI (*r* = 0.120, *P* = 0.248, Pearson’s correlation). The intestine may influence the absorption of TGs, but there was no difference in TG levels between CVID patients with (*n* = 26, median 1.42 mmol/l, IQR 0.74) and without (*n* = 69, median 1.28 mmol/l, IQR 0.74) enteropathy (*P* = 0.372).

Fifteen of the CVID patients (16%) had a history of cardiovascular disease (hypertension requiring medical treatment [*n* = 13], coronary artery disease [*n* = 2]), which does not appear to be increased compared to the general population (Supplemental Table 1). TG levels in these patients (*n* = 15, 1.45 ± 0.83 mmol/l) were not significantly different compared to patients without cardiovascular disease (*n* = 80, 1.23 ± 0.74 mmol/l), *P* = 0.650 (median ± IQR mmol/l). Of these 15 patients, six patients were using statins. We did, however, not find any significant difference in TG levels in statin users (1.46 ± 0.61 mmol/l) versus non-statin users (1.30 ± 0.76 mmol/l; *P* = 0.680), and when we excluded these six statin users from the analysis, the difference in TG levels between CVID (*n* = 89) and healthy controls (*n* = 28) was still significant (*P* = 0.010).

Since cardiovascular disease and dyslipidaemia constitute a greater risk in older than in younger adults, we further explored if the TG difference was related to age. First, within the CVID group, TG levels did not correlate with age (*r* = 0.177, *P* = 0.087, Pearson’s correlation). We then divided the CVID population and healthy controls into two groups: < 50 years and ≥ 50 years. These analyses showed that it was the younger CVID group that was driving the difference in TG between CVID and healthy controls, whereas in CVID patients ≥ 50 years, the difference in TG levels was not significant compared to controls (Table 2). In fact, the TG levels in CVID were numerically lower in CVID compared to healthy controls in the age group ≥ 50 years old. Importantly, however, very few controls were ≥ 50 years of age and the data should therefore be interpreted with caution.

**Table 2:** The difference in TG between CVID and healthy controls

Age in years	CVID	Healthy controls	*P*-value
CVID < 50, mean ± SD mmol/l (*n*)	1.24 ± 0.4 (51)	0.93 ± 0.4 (23)	0.009
CVID ≥ 50, mean ± SD mmol/l (*n*)	1.51 ± 0.6 (39)	1.79 ± 0.7 (5)	0.342

Data is from the *Main cohort* (95 CVID and 28 healthy controls).

### Triglycerides Correlate Positively with CRP, IL-6, and IL-12

**Fig. 2:**
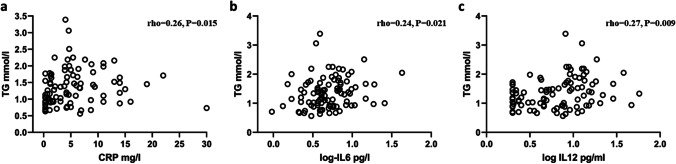
Correlation between TG levels and inflammatory markers in CVID. Panels show correlation between TG levels and CRP (**a**), IL-6 (**b**), and IL-12 (**c**) in 95 CVID patients. The IL-6 and IL-12 values were log transformed to improve visualization. Correlations were calculated by Spearman’s rank correlation test and are presented by rho. Data is from the Main cohort

Markers of systemic inflammation such as CRP, sCD25, sCD14, IL-6, IL-8, IL-12, and TNF are all elevated in CVID patients compared to controls [6]. Thus, we next examined if TG levels were associated with these inflammatory markers. We found that TG correlated significantly with CRP (rho = 0.256, *P* = 0.015), IL-6 (rho = 0.237, *P* = 0.021), and IL-12 (rho = 0.265, *P* = 0.009, Fig. 2A–C), but not with TNF (rho = 0.170, *P* = 0.099), sCD25 (rho = 0.098, *P* = 0.346), sCD14 (rho = 0.014, *P* = 0.894), or IL-8 (rho = 0.111, *P* = 0.284).

In contrast, TG levels did not correlate with IgG levels (rho = 0.180, *P* = 0.082), and we found no association between TG levels and T and B cell subpopulations (Supplemental Table S2).

### Triglycerides Correlate Strongly with the Gut Bacterial Toxin LPS

**Fig. 3:**
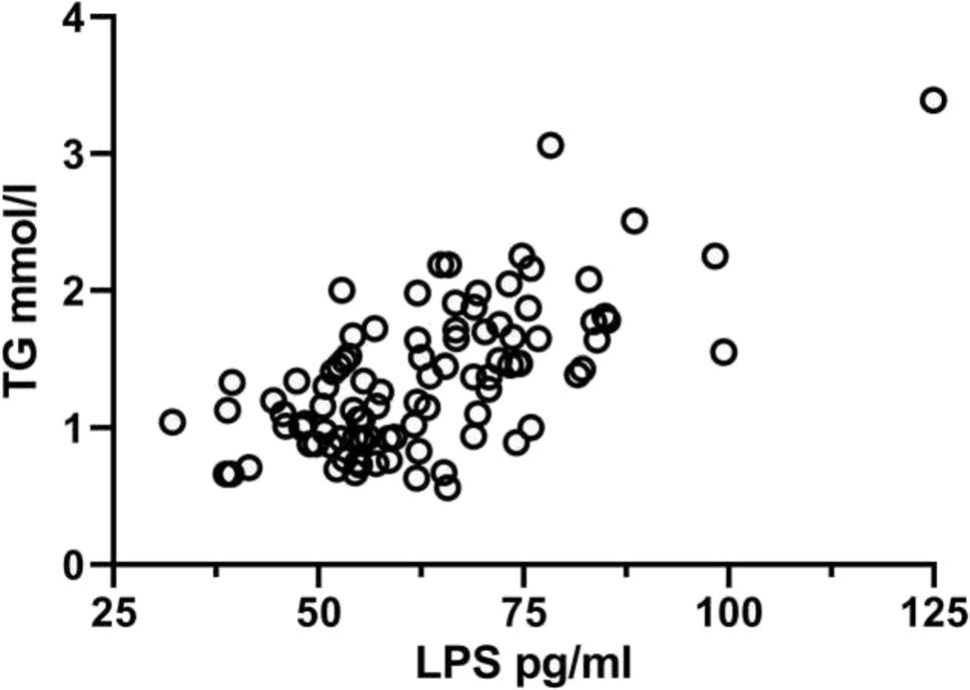
Correlation between TG levels and LPS in CVID. The figure shows correlation between TG levels and LPS in CVID patients (*n* = 95). Comparisons are made by Pearson’s correlation test (correlation coefficient *r*). Data is from the Main cohort

We have previously shown that CVID patients have increased plasma levels of the microbial toxin LPS, a marker of gut leakage, compared to controls, which has been associated with gut dysbiosis and markers of systemic inflammation [3, 12]. We therefore explored if TG concentrations correlated with LPS levels in CVID patients. As shown in Fig. 3, TG correlated strongly with LPS (*r* = 0.654, *P* = 6.59 × 10^−13^), suggesting a potential link between TG levels and gut microbial leakage.

### Longitudinal Analysis: Triglycerides Are Stable Over Time in CVID Patients

To explore if TG levels were stable over time, we measured TG levels in 20 CVID patients (mean age 51 years ± 12 [SD], 13 [65%] women) at three consecutive time points over 8 weeks. Sixteen patients completed all these three measurements (baseline, week 2, and week 8). Applying the Friedman test, we discovered no significant changes in TG levels (median TG levels ± IQR mmol/l in week 0: 1.06 ± 0.82, week 2: 1.22 ± 0.72, week 8: 1.16 ± 0.97; *P* = 0.269) (Supplemental Fig. S1).

### Plasma Triglycerides Correlate with the Gut Microbial CVID-Specific Dysbiosis Index

Next, we utilized the *Subset cohort* (*n* = 40), that included data from gut microbial analyses, lipoprotein profiling, FAs, and diet. We have previously shown that the microbial diversity (alpha diversity) in this *Subset cohort* is reduced compared to the healthy controls [28]. However, we did not find that TG levels correlate with alpha diversity, *r* =  − 0.236, *P* = 0.115 (PD-New) in this cohort. Alpha diversity is a universal microbial diversity measurement but is not disease specific. Next, we used the previously published CVID-specific dysbiosis index consisting of ten specific taxa (i.e., increased in CVID: *Bacilli*, *Dorea*, *Roseburia*, *Gammaproteobacteria*, and reduced in CVID: *Bifidobacterium*, *Odoribacteracea*, *Christensenellaceae*, *Blautia*, *Sutterella*, *Desulfovibrionacea*) found to capture the dysbiosis in stool samples from CVID patients [12]. A higher index value corresponds to a more unfavorable dysbiosis in the stool sample. We found that plasma TG levels correlated positively with the degree of gut dysbiosis in CVID, *r* = 0.315, *P* = 0.048. When correlating the ten taxa making up the CVID dysbiosis index individually to TG levels, we found that the bacterial class *Bacilli* correlated negatively with plasma levels of TG (rho =  − 0.36, *P* = 0.022, Table 3). These findings further support a link between TG and altered gut microbiota in CVID.

**Table 3:** Correlations between plasma, triglyceride levels, and bacterial taxa involved in gut microbial dysbiosis in CVID

	Increased in:	Correlation with TG	*P*-value
Dysbiosis index	CVID	0.315^a^	0.048
Bacterial taxa			
*Bacilli*	CVID	**− 0.360**^b^	**0.022**
*Dorea*	CVID	0.085^b^	0.601
*Roseburia*	CVID	0.094^b^	0.526
*Gammaproteobacteria*	CVID	0.111^b^	0.495
*Bifidobacterium*	Healthy	− 0.005^b^	0.976
*Odoribacteraceae*	Healthy	− 0.168^b^	0.301
*Christensenellaceae*	Healthy	− 0.110^b^	0.500
*Blautia*	Healthy	0.174^b^	0.283
*Sutterella*	Healthy	− 0.164^b^	0.311
*Desulfovibrionacea*	Healthy	0.248^b^	0.122

Significant bacterial taxa from Jørgensen et al., Mucosal Immunol 2016. *CVID*, common variable immunodeficiency

^a^Correlations were calculated by Pearson’s correlation test and are presented by Pearson’s *r*

^b^Correlations were calculated by Spearman's rank correlation test and are presented by rho

### Increased Levels of All Sub-fractions of VLDL Cholesterol in CVID

We performed lipoprotein profiling in the *Subset cohort* (*n* = 40) to explore, in detail, the lipid profile in CVID and its potential link to gut microbiota and diet. VLDL is the main transporter of TG from the liver to various tissues and, not surprisingly, we found a strong correlation between TGs in plasma and TGs in VLDL (*r* = 0.985, *P* = 1.92 × 10^−30^) in CVID patients. Moreover, lipoprotein profiling revealed significantly increased levels of all sizes of VLDL particles ranging from extra-large (XXL) VLDL to extra small (XS) VLDL in CVID patients compared to controls (Table 4). We have previously reported that CVID patients have similar LDL cholesterol levels to healthy controls [10]. Here, we extend these findings showing that this applies to all LDL subgroups (large, medium, and small LDL particles; Table 4).

**Table 4:** Overview of lipoproteins in CVID compared to healthy controls

Particle concentration	Phenotype	Mean value	*P*-value
XXL-VLDL	CVID	1.50 × 10^−10^	0.045^b^
	Controls	1.10 × 10^−10^	
XL-VLDL	CVID	7.08 × 10^−10^	0.028^b^
	Controls	4.61 × 10^−10^	
L-VLDL	CVID	4.62 × 10^−9^	0.046^a^
	Controls	3.11 × 10^−9^	
M-VLDL	CVID	1.68 × 10^−8^	0.038^a^
	Controls	1.28 × 10^−8^	
S-VLDL	CVID	2.72 × 10^−8^	0.014^a^
	Controls	2.18 × 10^−8^	
XS-VLDL	CVID	3.52 × 10^−8^	0.019^a^
	Controls	3.09 × 10^−8^	
IDL	CVID	9.50 × 10^−8^	0.417^a^
	Controls	9.10 × 10 − 8	
L-LDL	CVID	0.285	0.534^a^
	Controls	0.279	
M-LDL	CVID	1.24 × 10^−7^	0.522^a^
	Controls	1.19 × 10^−7^	
S-LDL	CVID	1.45 × 10^−7^	0.516^a^
	Controls	1.40 × 10^−7^	
Cholesterol	Phenotype	Mean value	*P*-value
TC	CVID	2.139	0.930^a^
	Controls	2.128	
VLDL-C	CVID	0.598	0.020^a^
	Controls	0.486	
LDL-C	CVID	1.400	0.704^a^
Triglycerides	Phenotype	Mean value	*P*-value
Triglycerides	CVID	1.251	0.021^a^
	Controls	0.999	
VLDL-triglycerides	CVID	0.863	0.029^a^
	Controls	0.651	
LDL-triglycerides	CVID	0.156	0.047^a^
	Controls	0.137	
HDL-triglycerides	CVID	0.127	0.494^a^
	Controls	0.122	

Data is from the *Subset cohort* (40 CVID and 28 healthy controls). *CVID*, common variable immunodeficiency; *HDL*, high-density lipoprotein; *LDL*, low-density lipoprotein; *VLDL*, very-low-density lipoprotein. The significant *P*-values are marked in bold as well as the phenotype that was significantly increased in either CVID or controls.

^a^Student’s *t*-test.

^b^Mann–Whitney *U* test.

### LPS Correlates with All Sizes of VLDL

As in the *Main cohort*, TG correlated strongly with LPS (*r* = 0.434, *P* = 0.005) also in the *Subset cohort*. Moreover, LPS correlated positively with the majority of the different sub-fractions of VLDL such as XXL-VLDL (rho = 0.553, *P* = 0.0003), M-VLDL (rho = 0.314, *P* = 0.048), S-VLDL (*r* = 0.432, *P* = 0.005), and XS-VLDL (*r* = 0.425, P = 0.006), but not XL-VLDL (rho = 0.251, *P* = 0.118) or L-VLDL (rho = 0.287, *P* = 0.073).

### Plasma Triglycerides Levels Correlated Inversely with a Favorable Fatty Acid Profile

TG molecules represent the major form of storage and transport of FAs in plasma and within cells. We have previously published that CVID patients have an unfavorable FA profile with reduced proportion of eicosapentaenoic acid (EPA) and docosahexaenoic acid (DHA) (two of the most abundant marine *n-3* polyunsaturated fatty acids [PUFAs] shown to have anti-inflammatory properties), and linoleic acid (LA) (the most abundant n-6 PUFAs), compared to healthy controls [11]. Recent studies support that LA may also have anti-inflammatory properties) [29]. By utilizing this dataset from the same individuals (*Subset cohort*), we found that TGs correlated negatively with DHA (rho =  − 0.369, *P* = 0.021) and LA (rho =  − 375, *P* = 0.019), but not with EPA (rho =  − 0.259, *P* = 0.111). These data may suggest a negative correlation between favorable, anti-inflammatory FAs and high levels of TGs.

### Triglycerides and All Sizes of VLDL Particles Did Not Correlate with Liver Function Tests

Since TGs are produced and assembled as VLDL particles in the liver, we explored whether liver function would affect TG or VLDL levels in CVID. Most of the CVID patients in the *Subset cohort* (*n* = 40) had normal liver tests (bilirubin: median 7 μmol/l [range 2–24], aspartate aminotransferase [AST]: median 27 U/l [min–max: 16–74], alanine aminotransferase [ALT]: median 27 U/l [min–max 9–49], and alkaline phosphatase [ALP]: median 77 U/l [min–max 45–255]). We found no correlations between TGs and bilirubin (rho = 0.046, *P* = 0.777), AST (rho = 0.046, *P* = 0.777), ALT (*r* = 0.272, *P* = 0.090), or ALP (rho = 0.164, *P* = 0.311). Likewise, there were no correlations between VLDL (total TG in VLDL) and bilirubin (rho = 0.098, *P* = 0.547), AST (rho = 0.043, *P* = 0.792), ALT (*r* = 0.267, *P* = 0.096), or ALP (rho = 0.079, *P* = 0.626).

### No Differences in Energy Intake in CVID Compared to Controls

**Fig. 4:**
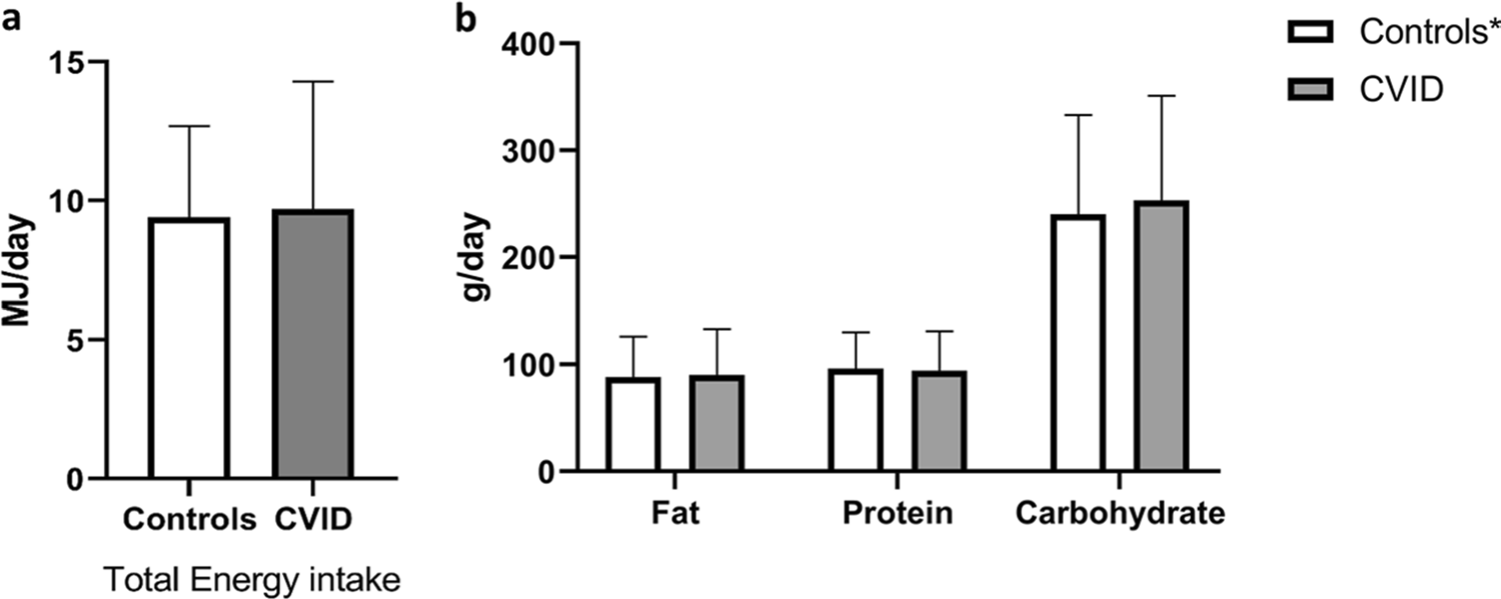
Dietary intake of energy, fat, protein, and carbohydrates in CVID and healthy controls. Daily intake of total energy intake (**a**) and fat, protein, and carbohydrates (**b**) in the CVID patient cohort (*n* = 38) versus the Norwegian background population (Norkost3, *n* = 1787)*. The Norkost 3 study used a 24-h recall questionnaire, aiding accuracy of reported diet, while using a high “*n*” to reduce the seasonal and day-to-day variation. Results are shown as mean and SD. *P > 0.05. Data is from the Subset cohort

Altered diet could potentially lead to increased VLDL and TG levels in CVID patients. We utilized the FFQ from the *Subset cohort*, filled in by 38 out of 40 CVID patients in this cohort. We found no differences in total energy consumption in megajoules per day in the CVID cohort (mean 9.7, SD ± 4.6) compared to the reference cohort (mean 9,4, SD ± 3.3), *P* = 0.692 (Fig. 4A). Likewise, there was no difference in the daily intake (g/day) of fat (90 ± 43 vs 88 ± 38, *P* = 0.68), protein (94 ± 37 vs. 96 ± 34, *P* = 0.72), or carbohydrates (254 ± 98 vs. 240 ± 93, *P* = 0.30) between CVID patients and the reference cohort, given in mean ± SD, respectively (Fig. 4B). Moreover, there were no significant correlations between TG or VLDL *and* the intake of total energy, fat, carbohydrates, protein, cholesterol, or saturated fat (Supplemental Table S3).

### The Presence of Bronchiectasis Does Not Affect TG or VLDL Levels in CVID

Finally, patients with bronchiectasis may have chronic infections or colonization of microbes, which may potentially influence TG and/or VLDL levels. In the *Main cohort*, 29 out of 95 patients (31%) had bronchiectasis (described on high-resolution computed tomography), and in the *Subset cohort*, 13 out of 40 patients (33%) had bronchiectasis. However, we did not find any difference in TG (1.41, [0.71] mmol/l vs. 1.23 [0.74] mmol/l, *P* = 0.202 [*Main cohort*]) or VLDL (1.38, [0.39] mmol/l vs. 1.08 [0.83] mmol/l, *P* = 0.283 [*Subset cohort*]) levels between CVID patients with or without bronchiectasis, respectively (values given in median [IQR]).

## Discussion

In this study, we present novel data to support the hypothesis that there is an interaction between lipid disturbances, systemic inflammation, gut leakage, and gut dysbiosis in CVID. Our main findings were (i) CVID patients had increased levels of TG and all particle sizes of VLDL; (ii) TG levels were associated with increased plasma levels of the inflammatory markers CRP, IL-6, and IL-12, and were higher in the clinical subgroup characterized by autoimmunity and organ-specific inflammation; (iii) both TG and VLDL were strongly associated with systemic LPS levels (iv); increased TG levels were associated with gut microbial dysbiosis in CVID patients and inversely correlated with a favorable FA profile;(v) no associations were found between TG and VLDL levels *and* diet (energy and fat intake), BMI, or cardiovascular disease/statin use in CVID patients.

HDL is known to possess anti-inflammatory properties, at least partly involving binding and neutralization of LPS [30], and we have previously showed that CVID patients have decreased levels and function of this “anti-inflammatory” lipoprotein [10]. In the current study, we expand the understanding of the metabolic profile in CVID patients and its relation to inflammation. We show that CVID patients also have increased levels of TG and all sub-fractions of VLDL compared to healthy controls. Moreover, TG levels were associated with raised systemic levels of inflammatory markers as well as the inflammatory CVID phenotype characterized by inflammatory and autoimmune complications. Of note, VLDL has previously been reported to induce an inflammatory response in monocytes, leading to enhanced monocyte/endothelial cell interaction [31]. TGs and TG-rich lipoproteins (i.e., VLDL) can also directly promote inflammatory responses in endothelial cells including induction of reactive oxygen species, involving activation of various transcriptional factors like NF*k*B [15]. Furthermore, TGs can develop toxic properties, subsequently activating the innate immune system via the NLRP3 inflammasome [32]. These VLDL/TG-mediated inflammatory responses seem to be enhanced by an unfavorable FA profile [31]. Notably, herein we found a negative correlation between TG levels and levels of DHA (*n-3* PUFA) and LA (*n-6* PUFA), both thought to mediate anti-inflammatory effects [11]. Thus, the present study shows that the lipid profile in CVID patients is not only characterized by decreased HDL levels, but also by increased levels of TGs and VLDL, all potentially contributing to the inflammatory phenotype in CVID patients.

In healthy individuals, more than 90% of plasma LPS has been shown to be lipoprotein bound, with highest binding capacity to HDL and the lowest to VLDL [33]. In the present study, we show a strong correlation between both TGs and VLDL *and* systemic LPS levels. The metabolic fate of LPS is highly dependent on the plasma lipoprotein balance [34, 35]. Thus, a shift to low HDL and high VLDL levels in CVID, as compared with healthy controls, could have influenced the increased LPS levels in CVID that were strongly associated with increased TG and VLDL levels. A positive correlation between LPS and TG has previously been shown in HIV infection and diabetes, two conditions that are also characterized by low-grade systemic inflammation [35–37]. Moreover, in patients with sepsis, LPS binds to VLDL instead of HDL, showing an increased affinity to lipoproteins with lower density during infection [38]. We hypothesize that a similar mechanism could occur in CVID, where systemic inflammation, decreased affinity to HDL or a combination thereof, induces LPS binding to switch from HDL to VLDL.

Several studies have found changes in gut microbiota composition in dyslipidemia, indicating a possible role of gut microbiota in the regulation of lipid metabolism [39]. TG levels have been associated with a reduction of gut microbial abundance (alpha diversity) in obese patients [40], with similar results in population studies after adjusting for BMI [41]. We have previously shown that alpha diversity is decreased in CVID compared to healthy controls [12, 28]. However, the CVID dysbiosis index is disease specific and different from other disease phenotypes, e.g., inflammatory bowel disease, as previously published [12]. A high value of the CVID dysbiosis index refers to a CVID-specific dysbiosis, whereas a low value of alpha diversity refers to a more general dysbiosis. Herein, we found an unfavorable lipid profile with increased TG and VLDL to be associated with the gut microbial–specific dysbiosis index in CVID, but alpha diversity did not correlate with TG levels in our study. The more dysbiosis in the stool samples from CVID patients, the higher TG levels measured in the blood, suggesting a role of gut microbiota in altered dietary fat absorption. Moreover, it is plausible that the altered gut microbial composition in CVID might fuel a possible interacting effect of LPS and lipids on systemic inflammation, as also discussed for HIV [36]. Our findings further support a link between disturbed lipid profile and gut dysbiosis in CVID, with inflammation as the most probable mediator. Data on microbial dysbiosis in other organs than the gut is limited in CVID [42]; particularly, there are no data on lower airway dysbiosis. We did not find any difference in TG levels in CVID patients with or without bronchiectasis in our cohort. However, as we lack microbiota data on the lower airways, we cannot rule out that subgroups of these patients could have airways dysbiosis that could potentially influence systemic inflammation and thereby TG/VLDL levels.

In the present study, we found no association between TG levels *and* gender, BMI, liver pathology, or diet, including energy and fat intake. Decreased HDL and increased TG/VLDL are often associated with a phenotype characterized by adiposity and increased occurrence of cardiovascular disease. This may not to be the case in CVID patients, as we found no association with BMI and TG levels. We estimated that 17% of the CVID patients had a diagnosis of cardiovascular disease, which does not appear to be increased compared to the general population. Moreover, these CVID patients showed no difference in TG levels as compared to CVID patients without cardiovascular disease and we found no association between statin use and TG levels. Interestingly, whereas age is risk factor for cardiovascular disease[43], it was the younger CVID patients that had higher TG levels than age-matched healthy controls, underscoring the important point of screening younger CVID patients. However, before any conclusion can be drawn in relation to the risk for cardiovascular disease in CVID, this important issue should be investigated in much larger cohorts, requiring international multi-center studies due to the rarity of CVID.

The association of TG with an inflammatory phenotype in the CVID patients may be of importance to explain the high levels of TG and VLDL in these patients. Thus, both LPS and several inflammatory cytokines inhibit degradation of TG by down-regulation of the enzyme lipoprotein lipase (LPL). ApoCIII is also involved in this process by inhibiting LPL and contributing to inflammatory effects of TG/VLDL. Therefore, we suggest that whereas high levels of TGs and VLDL in CVID patients may promote inflammation, inflammation may also promote these lipid disturbances, potentially representing a vicious inflammatory circle in these patients.

The strengths of this study are the inclusion of lipoprotein profiling, detailed dietary, and gut microbial data. The study also has some potential limitations. The fact that the samples were taken on non-fasting individuals could be considered a weakness of the study, but research also supports that non-fasting TG levels are more closely associated with relevant pathology than fasting TG levels [15]. Importantly, the controls were also non-fasting. Moreover, the longitudinal data on TGs confirmed stable TG levels over 8 weeks, implying that TGs are relative stable markers in this cohort. We did not have dietary data on the healthy controls and therefore used data from the National Dietary Survey as a control cohort for dietary analyses. Furthermore, it is difficult to estimate how large the proportion of patients with bronchiectasis had chronic infections or airway dysbiosis, and how this would have affected TG levels. The lack of these data as well as the lack of dysbiosis data from other organs such as the upper airways, are also limitations of this study. Many comparisons were performed; thus, some of the findings could be by chance. Finally, correlations do not necessarily prove any causal relationship, and the data from this study should be further examined in more mechanistic studies.

In conclusion, we show a disturbed lipid profile in CVID, with increased plasma levels of TGs and all sizes of VLDL, which is associated with systemic inflammation, LPS, and gut dysbiosis in CVID. TGs are particularly increased in CVID patients with inflammatory and autoimmune complications, and do not appear to be associated with diet, BMI, or the occurrence of cardiovascular disease. Future studies should explore the interplay between lipids, inflammation, gut leakage, and dysbiosis at a mechanistic level, and larger prospective studies are needed to fully elucidate the clinical consequences of altered lipid profile in CVID.

## Supplementary Information

Below is the link to the electronic supplementary material.

Supplementary file1 (DOCX 65 KB)

## Data Availability

The datasets analyzed during the current study are not publicly available due to Norwegian legislation regarding general data protection regulation but are available from the corresponding author (SFJ), on reasonable request.

## Abbreviations

BMI: Body mass index
CRP: C reactive protein
CVID: Common variable immunodeficiency
DHA: Docosahexaenoic acid
EPA: Eicosapentaenoic acid
FA: Fatty acid
FFQ: Food frequency questionnaire
HDL: High-density lipoprotein
Ig: Immunoglobulin
IL: Interleukin
LA: Linoleic acid
LDL: Low-density lipoprotein
LPS: Lipopolysaccharide
PUFAs: Polyunsaturated fatty acids
s: Soluble
TG: Triglycerides
TNF: Tumor necrosis factor
VLDL: Very-low-density lipoprotein
VLDL-TG: Total triglycerides in very-low-density lipoprotein
