# Supplementary material for: Increased Plasma Levels of Triglyceride-Enriched Lipoproteins Associate with Systemic Inflammation, Lipopolysaccharides, and Gut Dysbiosis in Common Variable Immunodeficiency
Source: J Clin Immunol. 2023 Mar 30;43(6):1229–40. doi: 10.1007/s10875-023-01475-x (PMC10353950; doi:10.1007/s10875-023-01475-x)
Supplement: Supplementary file 1 — Supplementary file1 (DOCX 65 KB) [file 10875_2023_1475_MOESM1_ESM.docx]

**Supplemental information**

**Title:**

**Increased plasma levels of triglyceride-enriched lipoproteins associate with systemic inflammation, lipopolysaccharides and gut dysbiosis in Common variable immunodeficiency**

**Supplemental methods**

*Gut microbiota analyses*

The sequencing was performed on an Illumina MiSeq. Briefly, the hypervariable V3-V4 region was amplified with generic primers as described in detail ^1^, including the gene specific primer sequences CCTACGGGAGGCAGCAG (forward) and GGACTACHVGGGTWTCTAAT (reverse) and up to 192 (24x8 barcodes) samples in parallel. Cleanup and normalization were performed using the SequalPrep Normalization Plate Kit (Life Technologies), followed by pooling and quality control. The final libraries were sequenced on an Illumina MiSeq (Norwegian Sequencing Centre, Oslo University Hospital Ullevål) using the v3 kit, allowing up to 300 basepairs paired-end sequencing. Paired-end reads were quality trimmed with cutadapt version 1.13 and then merged using FLASH version 1.2.11. The merged reads were de-multiplexed and quality filtered using default values in Quantitative Insights Into Microbial Ecology (QIIME) version 1.9.1. Closed reference operational taxonomic unit (OTU) mapping to the Silva database (version 123, reference OTUs clustered at 97% sequence similarity) was performed using SortMeRNA version 2.0 through QIIME. A rarefied OTU table (9525 reads per sample) was generated and OTUs with less than 2 reads to support it were discarded.

*Cohorts*

The *Main cohort* included CVID patients without ongoing acute infections. The *subset cohort* is the same cohort/samples as the baseline cohort in the previous published rifaximin study (n=40)^2^. The overall exclusion criteria for this cohort was: ongoing infection, antibiotics in the last 12 weeks, a history of allergic reaction to rifaximin, malignancy, impaired kidney function, pregnancy or lactation, immunosuppressive drugs, comorbidity that could influence with the patient’s safety or compromise the study results and polypharmacy (patients with an extensive medication list i.e. ten drugs or more [due to the anticipated effect on microbiota composition]). Three of these patients were on statins.

*Lipid measurements*

The lipoprotein subclass sizes were defined by their average diameter, as follows: extremely large (XXL) VLDL/chylomicrons (> 75 nm), extra-large (XL) VLDL (64 nm), large (L) VLDLL (53.6 nm), medium (M) VLDL (44.5 nm), small (S) VLDL (36.8 nm) and extra-small (XS) VLDL (31.3 nm), IDL (28.6 nm) and L LDL (25.5 nm), M LDL (23.0 nm) and S LDL (18.7 nm) subclasses. The TG and cholesterol components of the lipoprotein subclasses were also quantified. The mean size for VLDL and LDL particles was calculated by weighting the corresponding subclass diameters with their particle concentrations, as previously described^3^.

**Supplemental Tables**

**Supplemental Table S1. Comparison of TGs in CVID, patients stratified by B cell subclasses**

| **Subgroups** | **B cells:** | | **Transitional B cell**: | | **CD21^low^ B cells:** | | **Switched memory**  **B cells:** | |
| --- | --- | --- | --- | --- | --- | --- | --- | --- |
|  | ≤ 1%  (n=9) | >1 %  (n=79) | <9 %  (n=64) | ≥9%  (n=21) | <10 %  (n=54) | ≥10%  (n= 32) | ≤ 2%  (n=61) | >2%  (n=24) |
| **TGs mean**  **±SD** | 1.19  ±0.49 | 1.35  ±0.54 | 1.38  ±0.57 | 1.27  ±0.38 | 1.18  (0.75) | 1.34  (0.57) | 1.36  ± 0.52 | 1.31  ± 0.57 |
| **P value** | 0.40 | | 0.41 | | 0.93 | | 0.68 | |

B cells are classified according to EUROclass. Data were analyzed using Student’s t-test or Mann-Whitney *U* test, as appropriate. Values represent mean ± SD or ^a^median (IQR), as appropriate. CVID, common variable immunodeficiency; TGs, Triglycerides. Data is from the Main cohort.

**Supplemental Table S2. Correlation between TG, VLDL & VLDL-TG and the dietary intake of total energy, fat, carbohydrates, protein, cholesterol or saturated fat in CVID**

| **Dietary intake of** | **Correlation with *TG (rho)*** | **Correlation with *VLDL-TG (rho)*** |
| --- | --- | --- |
| Total energy, MJ/day | 0.060 | 0.055 |
| Fat, g/day | 0.0002 | -0.040 |
| Carbohydrates, g/day | 0.014 | 0.064 |
| Protein, g/day | 0.002 | -0.002 |
| Cholesterol, g/day | 0.031 | -0.037 |
| Saturated fat, g/day | 0.096 | -0.002 |

^a^Correlations were calculated by the Spearmans’ correlation test and are presented by rho. * p<0.05; **p<0.01; ***p<0.001.

CVID, common variable immunodeficiency; g, grams; MG, megajoules; TGs, Triglycerides; VLDL-TG, total TG in VLDL. Data is from the *Subset cohort*

**Supplemental Figure:**


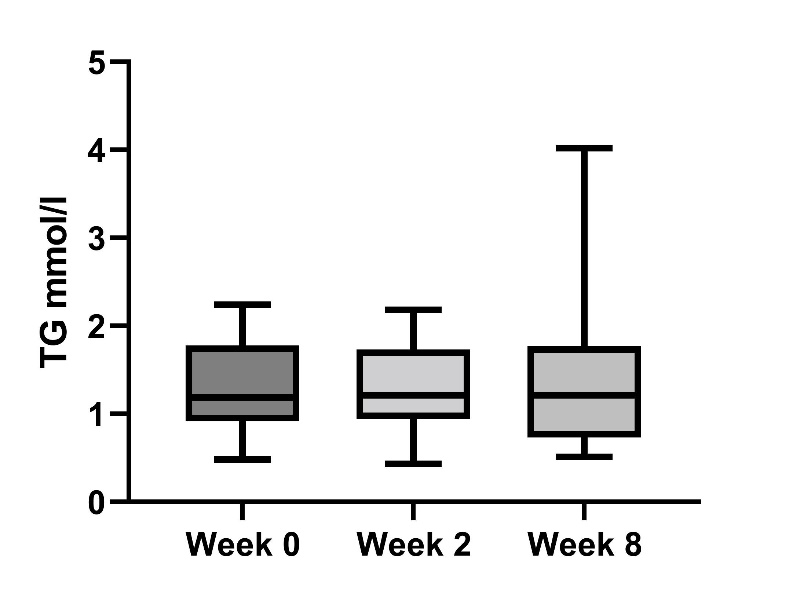
**Supplemental Figure S1:** Temporal testing of plasma levels of triglycerides (TG). Plasma TG levels over three time points (0, 2 and 8 weeks) in CVID patients (n=16). P-values were calculated using test or Friedman’s test. No significant differences were detected. Results are shown as mean with SD. TG, triglyceride.

**References:**

1 Kozich, J. J., Westcott, S. L., Baxter, N. T., Highlander, S. K. & Schloss, P. D. Development of a dual-index sequencing strategy and curation pipeline for analyzing amplicon sequence data on the MiSeq Illumina sequencing platform. *Appl. Environ. Microbiol.* **79**, 5112-5120, doi:10.1128/aem.01043-13 (2013).

2 Jørgensen, S. F. *et al.* Rifaximin alters gut microbiota profile, but does not affect systemic inflammation - a randomized controlled trial in common variable immunodeficiency. *Sci Rep* **9**, 167, doi:10.1038/s41598-018-35367-7 (2019).

3 Telle-Hansen, V. H., Christensen, J. J., Formo, G. A., Holven, K. B. & Ulven, S. M. A comprehensive metabolic profiling of the metabolically healthy obesity phenotype. *Lipids Health Dis.* **19**, 90, doi:10.1186/s12944-020-01273-z (2020).
